# Idiopathic AA amyloidosis presenting with initial abdominal pain: a case report and literature review

**DOI:** 10.3389/fmed.2025.1640436

**Published:** 2025-08-18

**Authors:** Pianpian Xia, Deliang Liu, Feihong Deng, Dalian Ou, Mingyang Deng

**Affiliations:** ^1^Department of Gastroenterology, The Second Xiangya Hospital, Central South University, Changsha, China; ^2^Research Center of Digestive Diseases, Central South University, Changsha, China; ^3^Clinical Research Center for Digestive Diseases in Hunan Province, Changsha, China; ^4^Department of Hematology, The Second Xiangya Hospital of Central South University, Changsha, China

**Keywords:** abdominal pain, AA amyloidosis, diagnosis, etiology, serum amyloid A protein, gastrointestinal diseases

## Abstract

Amyloidosis is a rare disease, often secondary to chronic inflammation or autoimmune disorders, with an unclear etiology in some cases. Herein, we report a 67-year-old male patient presenting with recurrent abdominal pain and multi-system involvement. The diagnosis of AA amyloidosis was confirmed by Congo red staining of small intestinal mucosal and bone marrow biopsies. Despite comprehensive screening, no definite etiology was identified. This case highlights that amyloidosis should be considered in patients with unexplained abdominal pain and multisystem abnormalities, and early tissue biopsy is crucial for diagnosis.

## 1 Introduction

Amyloidosis encompasses a group of disorders characterized by protein misfolding resulting from diverse etiologies. These misfolded proteins aggregate into amyloid fibrils with a β-sheet structure and abnormally deposit in the extracellular matrix, thereby impairing the structure and function of tissues and organs (1). Under electron microscopy, these proteins display a characteristic fibrillar morphology, consistent with their β-sheet structure. Diagnosis is confirmed when affected tissue, following Congo red staining, exhibits the characteristic apple-green birefringence under polarized light (2). Amyloidosis can involve virtually all organs. To date, over 42 distinct human proteins have been identified to form amyloid, yet only a small number are encountered clinically (3). The current nomenclature is based on the precursor protein that aggregates to form amyloid. The first letter “A” denotes amyloid, with the subsequent letter indicating the precursor protein (4). Amyloidosis can be divided into two main types according to organ involvement: systemic and localized. Etiologically, it is categorized into primary amyloidosis (AL), secondary amyloidosis (AA), hereditary amyloidosis, senile amyloidosis, and dialysis-related amyloidosis. In primary amyloidosis (AL), the precursor proteins originate from monoclonal immunoglobulin light chains (λ and κ variants). Secondary amyloidosis (AA) typically arises from chronic inflammatory or autoimmune disorders, with its precursor protein originating from fragments of the acute-phase reactant serum amyloid A (SAA). The most prevalent amyloid protein in hereditary and senile amyloidosis is transthyretin (TTR), while dialysis-related amyloidosis primarily arises from impaired clearance and subsequent accumulation of β_2_-microglobulin (β_2_-MG) *in vivo* (5). Primary amyloidosis is relatively the most common subtype, typically associated with plasma cell dyscrasias (6).

Amyloidosis is a rare disease. Recent data estimate the global incidence of AL amyloidosis at 10 cases per million people, with a 20-year prevalence of 51 cases per million (7–9). AA amyloidosis is relatively rare, and GI amyloidosis is even less common (10). Hagen et al. (11) statistically analyzed 2,511 GI amyloid specimens typed by proteomics-based methods from 2008 to 2021 and found that the main type of GI amyloidosis was AL type, followed by ATTR type, and AA type accounted for only 5%. The vast majority of GI amyloidosis lacks overt clinical manifestations, whereas symptomatic GI amyloidosis is exceedingly rare (12). Meanwhile, most patients with symptomatic GI involvement present with motility disturbances, malabsorption, or bleeding (13). Clinical manifestations vary based on the type, location, and extent of fibril deposition. Delayed diagnosis is common due to limited clinical awareness among clinicians (14).

According to the existing literature, there are few reports of AA amyloidosis cases presenting initially with abdominal pain and without a definite underlying cause. This case provides new insights into clinical diagnosis and management, aiming to deepen understanding of the complex gastrointestinal manifestations of systemic amyloidosis and improve recognition and management of the rare AA subtype.

## 2 Case

**FIGURE 1:**
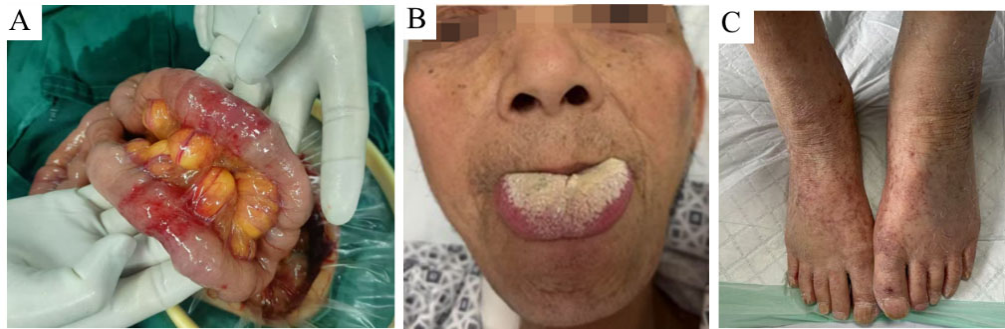
Intraoperative photographs of the small intestine and clinical signs. **(A)** Extensive and diffuse granulomatous-like edema and thickening of the small intestine observed during surgery. **(B)** Macroglossia noted in the patient. **(C)** Multiple scattered ecchymoses on the extremities.

A 67-year-old male patient presented to our hospital on 30 December 2024, with a 2-month history of abdominal pain that had worsened over the preceding 2 weeks. The patient initially presented with intermittent, cramp-like abdominal pain predominantly localized to the left lower quadrant, which commenced in early November 2024. The pain resolved spontaneously within minutes and was accompanied by borborygmi and abdominal distension, with no association with ingestion or defecation. Furthermore, the patient reported limb numbness and scattered ecchymoses involving the extremities. No symptoms of vomiting, obstipation, absence of flatus, melena, hematochezia, acid reflux, or heartburn were reported. The patient initially did not seek medical attention and intermittently used analgesics for self-management. On 3 December 2024, the patient presented to a local hospital due to recurrent abdominal colic while cycling. Abdominal CT showed diffuse small bowel wall thickening and ascites. The initial impression was acute diffuse peritonitis with suspected GI perforation. On 18 December 2024, the patient underwent emergency laparotomy. Intraoperatively, approximately 200 ml of dark red bloody fluid was found, but no GI tract perforation was identified. Granulomatous-like edematous thickening was observed extending from the ileocecal region to the terminal ileum (Figure 1A). Postoperatively, the patient was administered anti-infective therapy, but severe abdominal pain persisted during hospitalization. The patient experienced frequent yellow loose stools (three times daily) and had lost 15 kg in the prior 2 months.

**FIGURE 2:**
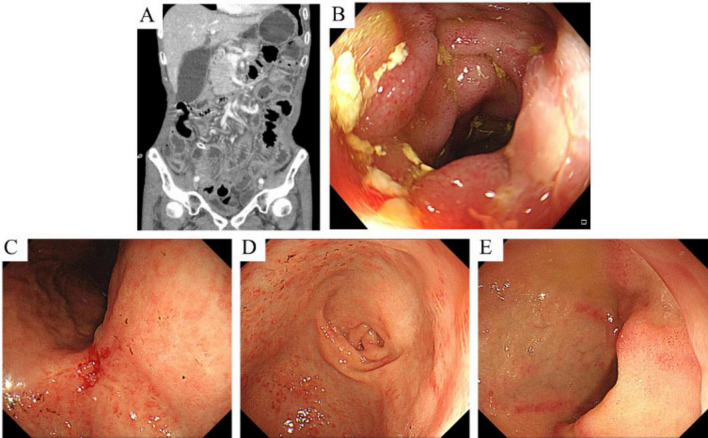
CT enterography (CTE) images and endoscopic findings of the small intestine. **(A)** CTE shows diffuse edema of the small intestinal wall with slight dilation of the lumen and associated gas and fluid accumulation. **(B)** Endoscopy reveals multiple patchy erosions and irregular ulcers at the terminal ileum. **(C,D)** Endoscopy shows mucosal redness and swelling in the gastric angle and antrum, along with a scar-like lesion on the posterior wall near the gastric angle, surrounded by swollen tissue and mucosal convergence. **(E)** Endoscopy shows multiple hyperemic erosions and ulcerations in the descending part of the duodenum.

Upon admission, physical examination confirmed macroglossia (Figure 1B) and multiple petechial purpuric lesions on the extremities (Figure 1C). Laboratory results showed hemoglobin at 80 g/L (reference: 130–175 g/L); urine analysis revealed 284.80 red blood cells/μl, 107.60 white blood cells/μl, and 2+ proteinuria, with 24-h urinary protein excretion at 826.4 mg/day (reference: <150 mg/day); fecal occult blood was positive; serum albumin was low at 24.5 g/L (reference: 40–55 g/L); liver enzymes were elevated—gamma-glutamyl transpeptidase at 145.0 U/L (reference: 10–60 U/L) and alkaline phosphatase at 126.5 U/L (reference: 45–125 U/L); creatinine was high at 192.0 μmol/L (reference: 44–133 μmol/L), indicating renal dysfunction; cardiac biomarkers were increased—myoglobin at 345.4 μg/L (reference: 25–58 μg/L) and high-sensitivity troponin T at 18.20 pg/ml (reference: 0–14 pg/ml)—along with elevated N-terminal pro-brain natriuretic peptide at 859.0 pg/ml (reference: 0–125 pg/ml); thyroid tests indicated hypothyroidism, with low free triiodothyronine (2.8 pmol/L; reference: 3.5–6.5 pmol/L) and free thyroxine (10.9 pmol/L; reference: 11.5–22.7 pmol/L), and high-sensitivity thyroid-stimulating hormone at 18.44 μIU/ml (reference: 0.55–4.78 μIU/ml); systemic inflammation was evident through markedly elevated erythrocyte sedimentation rate (44 mm/h; reference: 0–15 mm/h), C-reactive protein (166.40 mg/L; reference: 0–6 mg/L), procalcitonin (0.853 ng/ml; reference: 0–0.05 ng/ml), and interleukin-6 (52.2 pg/ml; reference: 0–7 pg/ml). Cardiac ultrasound showed a dilated ascending aorta, impaired left ventricular wall motion coordination, mild aortic valve degeneration with regurgitation, and mild tricuspid regurgitation. Pulmonary artery systolic pressure was estimated at 37 mmHg, with normal left ventricular systolic function. Small intestine CT enterography (CTE) revealed diffuse wall edema, mild luminal dilation, and gas-fluid levels (Figure 2A). Colonoscopy showed severe mucosal congestion and edema in the terminal ileum, with multiple erosions and irregular ulcers; whereas the colonic mucosa appeared normal (Figure 2B). Gastroscopy found diffuse congestion and edema in the gastric (Figures 2C, D) and duodenal mucosa (Figure 2E), along with scattered erythema and a scar-like lesion on the posterior gastric angle wall (Figure 2C). Based on the comprehensive evaluation of clinical history, physical findings, lab results, imaging, endoscopy, and surgery, amyloidosis, or other systemic diseases were considered in the differential diagnosis.

**FIGURE 3:**
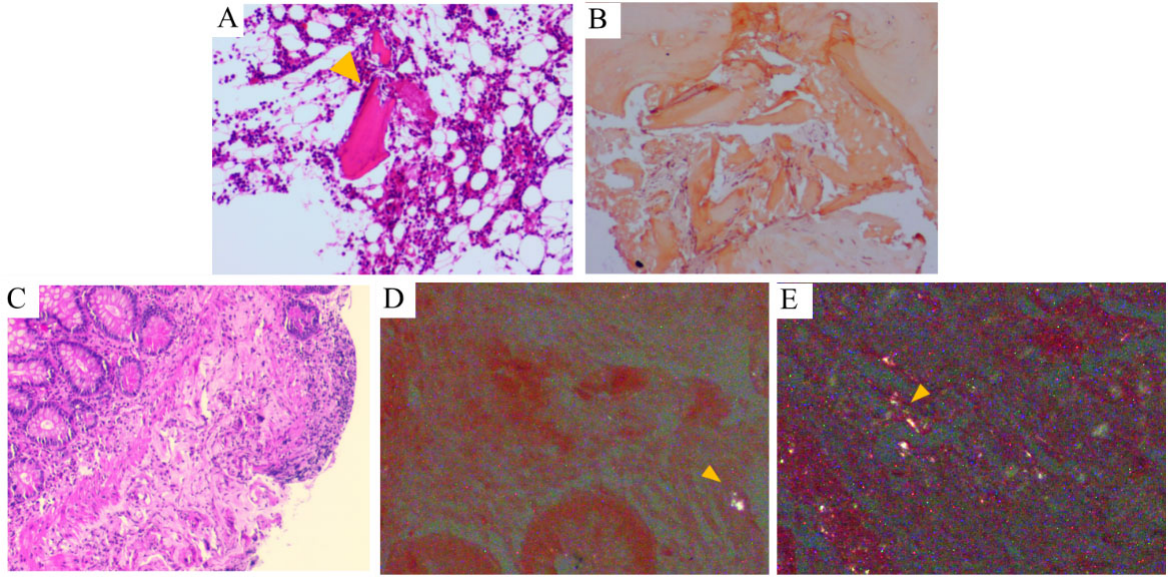
Histological findings of bone marrow and terminal ileum. **(A)** Bone marrow biopsy reveals pink, amorphous, hyaline material within the bone marrow tissue (HE, ×100). **(B)** Congo red staining under polarized light microscopy demonstrates amyloid deposition in the bone marrow, characterized by apple-green birefringence (×100). **(C)** Terminal ileum biopsy shows mildly disordered villous architecture with partial erosion (HE, ×100). **(D,E)** Submucosa of the terminal ileum contains pink, amorphous, hyaline material that exhibits apple-green birefringence under polarized light microscopy (arrows indicate positive areas).

Further investigations were conducted, including comprehensive analysis of blood and urine Bence Jones protein, blood and urine immunofixation electrophoresis, serum free light chain measurement, vascular endothelial growth factor (VEGF) assessment, immunoglobulin panel testing, connective tissue-related antibody screening, and bone marrow biopsy. All results were negative. Cardiac MRI showed minimal myocardial fibrosis in the left ventricular lateral wall, with no significant thickening. Electromyography confirmed active peripheral neuropathy in the limbs. Notably, histopathological evaluation of the bone marrow biopsy confirmed the presence of amyloid deposition, as indicated by Congo red staining exhibiting apple-green birefringence under polarized light within the medial aspect of the Haversian canals of the bone (Figures 3A, B). Likewise, histopathology of ileal biopsies confirmed amyloid deposition via positive Congo red staining and apple-green birefringence under polarized light (Figures 3C–E). Following multidisciplinary review, the diagnosis of systemic amyloidosis was confirmed. Although bone marrow smear analysis showed a slight increase in plasma cell proportion (8%), bone marrow biopsy revealed no significant abnormal plasma cell proliferation. Furthermore, bone marrow biopsy demonstrated weak positivity for both κ and λ light chains without obvious proportional imbalance, lacking the typical features of monoclonal plasma cells. Since no evidence of plasma cell clonality or κ/λ light chain proportional abnormalities was identified in blood/urine Bence Jones protein, blood/urine immunofixation electrophoresis, serum free light chain measurement, bone marrow biopsy, or bone marrow immunohistochemistry, AL amyloidosis was excluded, and the slight elevation of plasma cells in the bone marrow smear was deemed reactive. Accordingly, bone marrow FISH testing was not performed. ATTR amyloidosis was also deemed unlikely due to disease duration over 2 months and limited cardiac involvement. The patient was ultimately diagnosed with AA amyloidosis affecting multiple organs, including the small intestine, kidneys, liver, nerves, skin, thyroid, and possibly the heart. However, no etiological factors such as chronic infection, autoimmune disease, obesity, or tuberculosis were found. Therefore, idiopathic AA amyloidosis was considered.

During hospitalization, the patient received anti-infective therapy with cefoperazone and ornidazole, along with albumin supplementation, intestinal flora modulation, fluid replacement, and symptomatic supportive care. These therapeutic measures resulted in a significant reduction in abdominal pain. On 14 January 2025, following the administration of a 300 mg intravenous infusion of infliximab (IFX) for inflammatory control, the patient was discharged. After discharge, the patient remained asymptomatic with no abdominal pain or diarrhea. On 28 January 2025, the patient received the second cycle of IFX.

## 3 Discussion

### 3.1 GI involvement characteristics of AA amyloidosis

Amyloidosis most frequently involves the heart, whereas GI involvement is relatively uncommon. Among amyloidosis subtypes associated with GI pathology, AL amyloidosis is the most prevalent, followed by ATTR and AA amyloidosis (12, 15). It is noteworthy that the kidney, rather than the GI tract, represents the primary target organ in AA amyloidosis. Moreover, although GI symptoms may serve as the initial clinical presentation in various forms of amyloidosis, biopsy-confirmed deposition of amyloid proteins within the GI tract remains a relatively rare occurrence (12, 15, 16). In the case presented in this study, the patient manifested gastrointestinal symptoms complicated by multi-organ involvement (kidneys, liver, nerves, skin, thyroid, and suspected cardiac involvement), and was ultimately diagnosed with systemic amyloidosis based on positive findings from gastrointestinal tissue biopsies. Given that comprehensive clinical evaluations failed to identify evidence of abnormal plasma cell clonality and lacked diagnostic criteria for AL amyloidosis associated with plasma cell dyscrasias (17, 18), the more common AL subtype in gastrointestinal amyloidosis was excluded, and the patient was diagnosed with AA amyloidosis. This is extremely rare in clinical practice.

GI amyloidosis predominantly affects elderly male patients (19), with the small intestine representing the most commonly involved anatomical site (20), which is consistent with this patient. Previous observational studies have demonstrated that the most prevalent clinical manifestations of amyloidosis involving the GI tract include diarrhea, abdominal pain, anorexia, and GI bleeding (15, 21). In addition, Fernandes et al. (22) reported a rare case of primary systemic amyloidosis presenting as severe acute liver failure, highlighting the importance of considering amyloidosis in the differential diagnosis of unexplained liver dysfunction. The authors further identified three key radiological features of hepatic involvement in systemic amyloidosis on CT and MRI: hepatomegaly, liver heterogeneity, and particularly, periportal vein involvement. Our statistical analysis, based on data from Supplementary Table 1, demonstrates that among cases presenting with GI tract symptoms as the initial clinical manifestation and ultimately diagnosed with AA amyloidosis, diarrhea is the most frequently observed symptom (11 cases) (23–33), followed by abdominal pain (8 cases) (23, 29, 34–40) and GI bleeding (6 cases) (24, 34, 35, 38, 41, 42). These findings are consistent with previously published evidence. Notably, the clinical management of AA amyloidosis predominantly focuses on reducing proteinuria and maintaining or improving renal function. It should be emphasized that GI involvement in amyloidosis frequently manifests with non-specific clinical features. These symptoms often closely resemble those observed in more common GI diseases, which usually leads to diagnostic confusion. As a result, establishing an accurate diagnosis of GI amyloidosis remains a major challenge in clinical practice. Enhancing clinicians’ awareness of this condition—particularly its relatively rare AA subtype—is critical for facilitating early detection and effective therapeutic intervention.

### 3.2 Endoscopic manifestations of GI amyloidosis

Endoscopy with biopsy is essential for diagnosing amyloidosis affecting the GI tract. GI amyloidosis can present with a variety of mucosal abnormalities, such as edema, loss of mucosal luster, irregularities with nodules, thickened and scalloped valve nodules, erosions, ulcers, fragility, narrowing, and mucosal or submucosal hematomas (43). Previous studies indicate that the endoscopic features of GI amyloidosis are associated with amyloid protein deposition in the digestive tract. Different amyloid proteins show distinct deposition patterns: AL, Aβ2MG, and ATTR tend to accumulate in the submucosa or muscularis propria, whereas AA is more commonly found in superficial layers such as the mucosa or submucosa (44–46). In familial amyloid polyneuropathy, although amyloid deposition is minimal, it preferentially affects the GI nerves. Pathologists found that the amyloid protein was mainly located in the mucosal lamina propria in this case, confirming AA amyloidosis. Previous reports describe fine-grained and fragile mucosa as typical endoscopic features of AA amyloidosis. In this patient, multiple ulcers and mucosal fragility were observed in the terminal ileum, consistent with earlier findings. However, endoscopic findings in GI amyloidosis may appear normal or lack specific changes (47). This suggests that GI amyloidosis lesions are often non-specific. Clinicians should consider amyloidosis when detecting unexplained mucosal changes during endoscopy and be more willing to perform biopsies with Congo red staining.

### 3.3 Application of biologics in the treatment of AA amyloidosis

In recent years, there has been increasing evidence of the potential application of biologics in AA amyloidosis (48). In Supplementary Table 2, we summarized all case reports (Supplementary Table 2a) (49–84) and observational studies or systematic reviews (Supplementary Table 2b) (85–97) on the treatment of AA amyloidosis with biologics retrieved from PubMed.

We found that the main types of biologics currently applied to AA amyloidosis include anti-TNFα agents [IFX (57, 61, 67, 72, 73, 84), adalimumab (56, 98, 99), and etanercept (54, 66, 68, 79)], anti-IL-1 agents [anakinra (49, 51, 52, 58, 64, 69, 71, 75, 80, 81) and canakinumab (59, 77)], and anti-IL-6 agents [[tocilizumab (53, 55, 60, 62, 63, 74, 76, 78, 82, 83)]. There is also limited evidence indicating the successful application of IL-12/23 agents (50), JAK agents (65), and CD80/CD86-CD28 costimulatory pathway agents (70) in AA amyloidosis. The vast majority of the evidence reports the efficacy of biologics as first-line treatment, and some literature also reports successful cases of biologics as second-line (50, 53, 55, 56, 62, 80, 82) and fourth-line (65) treatments, indicating that biologics have positive treatment effects in patients with different treatment difficulties. However, we also found that selecting biologics for patients with AA amyloidosis should align closely with their underlying diseases. For example, in those with Crohn’s disease as the primary condition, biologics such as IFX, adalimumab, and ustekinumab—commonly used to manage inflammatory bowel disease—are often prescribed (50, 56, 61, 72). On the other hand, anakinra is typically selected for cases secondary to Familial Mediterranean Fever (FMF) (51, 52, 69, 71, 75, 80, 84). It is worth noting that 6%–19% of AA amyloidosis cases have unknown causes (100), and the number of such idiopathic cases is on the rise (101). In this case, our medical team systematically evaluated potential factors related to AA amyloidosis, including chronic infections, inflammation, tumors, and family history. After a thorough analysis, no definitive cause of amyloidosis was identified in this patient, leading to a diagnosis of idiopathic AA amyloidosis. This highlights gaps in our current understanding of its underlying pathophysiological mechanisms. To achieve more refined and precise management of this disease, in-depth exploration of its pathogenesis is urgently needed in the future. Currently, only three articles have reported the effects of biologics in treating AA amyloidosis of unknown etiology. Two of them used IL-1 agents [anakinra (89, 102) and canakinumab (102)], and the other used tocilizumab (88). All of them showed that biologics could significantly reduce SAA levels. However, the existing evidence has limitations. Firstly, the follow-up duration for most biologics applications is less than 1 year (50, 53, 56, 57, 59, 60, 64, 66, 71, 72, 74–76, 78, 80, 83, 98, 99). Secondly, individual cases show that failure may occur after extended follow-up (52, 68). Finally, not all of the existing evidence demonstrates the positive treatment effects of biologics (50, 64, 72). Therefore, large-scale clinical studies are needed in the future to evaluate the long-term safety and effectiveness of these biologics.

Supplementary Table 2b summarizes the existing observational studies and systematic reviews on the efficacy of biologics in AA amyloidosis (85–97). We found that these studies mainly focus on inflammatory arthritis (86, 87, 91, 92, 94) and FMF (90, 96, 97), and are mainly observational reports on TNF-α agents (85–87, 91–94). Overall, the cohort study by Esatoglu et al. (85) has a relatively large sample size and a long follow-up time. Its results show that TNF-α agents can successfully control AA amyloidosis caused by various factors, including inflammatory arthritis such as rheumatoid arthritis and ankylosing spondylitis. In 2016 and 2025, there were respectively a literature comprehensively evaluating the effectiveness and safety of AA amyloidosis secondary to FMF. The 2016 report focused on IL-1 agents (97), and the 2025 report on IL-6 agents (90). These two articles show that IL-1 and IL-6 agents seem to be a safe and effective alternative therapy for FMF patients who do not respond to or cannot tolerate colchicine.

In our case, the biologic selected for the patient was IFX, which is the first case of applying IFX to AA amyloidosis of unknown etiology. This choice was mainly based on the patient’s typical GI symptoms and economic ability. Currently, the patient is still under follow-up, and we look forward to new feedback on treatment effects.

## 4 Conclusion

This case indicates that AA amyloidosis can have an insidious onset and an unknown cause. Clinicians need to be more vigilant about multi-system involvement. Early tissue biopsy (such as small intestinal mucosa and bone marrow) combined with Congo red staining is the key to diagnosis. In the future, further exploration of the long-term efficacy of biologics and the molecular mechanisms of AA amyloidosis of unknown etiology is required.

## Data Availability

The original contributions presented in this study are included in this article/Supplementary material, further inquiries can be directed to the corresponding author.
