# Supplementary material for: Idiopathic AA amyloidosis presenting with initial abdominal pain: a case report and literature review
Source: Front Med (Lausanne). 2025 Aug 18;12:1640436. doi: 10.3389/fmed.2025.1640436 (PMC12399618; doi:10.3389/fmed.2025.1640436)
Supplement: Supplementary file 1 [file Table_1.docx]

## Supplementary Tables

Table 1 Summary of Case Characteristics in Patients with AA Amyloidosis Initially Presenting with GI Symptoms

| **#** | **Authir（Year）** | **GI Symptom** | **Amyloid Typing** | **GI biopsy site** | **Etiology** |
| --- | --- | --- | --- | --- | --- |
| 1 | Akharume, O., et al. (2024). | Initial bloody stools, followed by rectal bleeding | AA | Colon, rectum, kidney | Secondary to chronic skin and soft tissue infections in injecting drug users |
| 2 | Setake, M., et al. (2024). | Anorexia | AA | Stomach | Rheumatoid arthritis |
| 3 | Endo, H., et al. (2023). | Severe diarrhea | AA | Intestine | Inflammatory hepatocellular adenoma |
| 4 | Park, H. M., et al. (2023). | Epigastric pain, bloody stools | AA | Stomach | Infection with SARS-CoV-2 |
| 5 | Lacetera, R., et al. (2022). | Abdominal pain, elevated liver enzymes | AA | Kidney | Primary Sclerosing Cholangitis |
| 6 | Tanaka, T., et al. (2021). | Refractory diarrhea | AA | Ileum | Chronic pyelonephritis |
| 7 | Hashmi, S., et al. (2021). | Abdominal pain and rectal bleeding | AA | Gallbladder, gastric body, rectum, duodenum, and kidney | Possibly related to the presence of gout, obesity, and a heterozygous complex variant of the familial Mediterranean fever gene |
| 8 | Huang, Y., et al. (2020). | Nausea, hematemesis, diffuse abdominal pain | AA | Stomach | Chronic infection |
| 9 | Matsuda, S., et al. (2019). | Right upper quadrant pain and fever | AA | Gallbladder | Acute cholecystitis |
| 10 | Galmiche, S., et al. (2019). | Fatigue, anorexia, diarrhea | AA | Duodenum | Rheumatoid arthritis |
| 11 | Calderaro, J., et al. (2018). | Diarrhea, rectal bleeding | AA | Kidney, liver, rectum, colon, spleen, thyroid, ovary, adrenal gland | NA |
| 12 | Park, S. W., et al.(2018) | Nausea, vomiting, and abdominal pain | AA | Duodenum | Ulcerative colitis |
| 13 | Olmos-Martínez, J. M., et al. (2017). | Chronic diarrhea | AA | Colon | Jugular paraganglioma |
| 14 | Raghunathan, V., et al. (2017). | Abdominal pain, abdominal distension, and intestinal gas | AA and ATTR | Colon | Not clearly defined; the patient had multiple myeloma |
| 15 | Frommer, D. J., et al. (2014). | Main symptoms included diarrhea, with other symptoms including bleeding, vomiting, and abdominal pain | Four cases of AA | Intestine | Infection |
| 16 | Kato, T., et al. (2012). | Elevated liver enzymes | AA | Salivary gland and stomach | Primary sclerosing cholangitis |
| 17 | Baird-Howell, M. A. and J. Wurzel (2011). | Massive GI bleeding | AA | GI tract | Recurrent infections |
| 18 | McMahan, Z. H., et al. (2010). | Chronic diarrhea | AA | Small intestine | Ankylosing spondylitis |
| 19 | Brünnler, T., et al. (2009). | Diarrhea, recurrent lower abdominal pain | AA | Colorectum | Neutropenia |
| 20 | Sýkora, R., et al. (2008). | Diarrhea, weight loss, and circulatory failure | AA | Kidney | NA |
| 21 | Fushimi, T., et al. (2005). | Diarrhea | AA | Intestine | Rheumatoid arthritis |
| 22 | Koloktronis, A., et al. (2003). | Difficulty chewing and swallowing food | AA | Rectum, tongue | Psoriatic arthritis, ankylosing spondylitis |
| 23 | Jadoul, M., et al. (2001). | Abdominal pain and diarrhea | AA | Initially kidney, then rectum, gallbladder, thyroid | TRAPS, genetic variation |

GI: Gastrointestinal, TRAPS: TNF receptor associated periodic syndrome, NA: Not Available.

Table 2a Efficacy of Biologic Agents in the Treatment of AA Amyloidosis: Case Report

| **#** | **Author(Year)** | **Age/Sex** | **Biologics** | **Type of biologics** | **Biopsy site** | **Etiology** | **Duration OF FOLLOW-UP** | **Treatment OUTCOMES** |
| --- | --- | --- | --- | --- | --- | --- | --- | --- |
| 1 | Hassani, K., et al. (2018). | 34/F | IFX | TNFα biologics | Kidney | CD | 24 months | Renal function remained normal,  Proteinuria decreased, No side effects. |
| 2 | Kuroda, T., et al. (2008). | 55/F | IFX | TNFα biologics | Kidney, GI mucosa | RA | 18 months | Clinical symptoms relieved,  Proteinuria decreased significantly,  eGFR59.0 mL/min/1.73 m2,  Serum creatinine 0.7 mg/dL,  Amyloid deposition significantly resolved,  SAA and CRP significantly decreased to low levels. |
| 3 | Pukitis, A., et al. (2013). | 24/M | IFX | TNFα biologics | Kidney | CD | 3 months | Clinical symptoms relieved,  CRP and SAA decreased significantly,  Serum creatinine increased significantly and eGFR decreased rapidly to renal failure |
| 4 | Fiehn, C., et al. (2003) | 64/F | IFX | TNFα biologics | Kidney | Psoriatic arthritis | 7 months | Clinical symptoms relieved,  Renal function recovery,  Proteinuria decreased. |
| 5 | Yüksel, S., et al. (2003) | 12/F | IFX | TNFα biologics | Kidney, rectum, and duodenum | FMF | 22 months | Clinical symptoms improved,  CRP decreased to normal level,  Creatinine slightly elevated to 1.1mg/dL,  Proteinuria decreased significantly to 45mg/m^2^/h. |
| 6 | Fikri-Benbrahim, O., et al. (2013) | 57/F | ADA | TNFα biologics | Abdominal subcutaneous fat | RA | 12 months | Decreased proteinuria |
|  |  | 78/F | ADA | TNFα biologics | Abdominal subcutaneous fat | RA | NA | Low blood pressure levels and progressive deterioration of renal function occur,  Multiple episodes of volume overload and decompensation of baseline heart disease |
| 7 | Nowak, B., et al. (2009) | 31/F | ADA | TNFα biologics | Kidney | polyarticular-onset RF-negative ANA-positive juvenile chronic arthritis | 4 months | Clinical symptoms relieved,  Renal function improved,  Decreased proteinuria. |
| 8 | Drewe, E., et al. (2004). | 27/F | etanercept | TNFα biologics | Kidney and spleen | TRAPS | 24 months | Clinical symptoms relieved,  Proteinuria decreased,  eGFR improved to 59 mL/min/1.73 m2,  SAA decreased to less than 10 mg/L. |
|  |  | 54/M | etanercept | TNFα biologics | Kidney, spleen, liver | TRAPS | 25 months | eGFR improved to 83mL/min/1.73m2,  Stability of SAA |
| 9 | Kobak, S., et al. (2007). | 47/F | etanercept | TNFα biologics | Rectum, minor salivary glands | AS | 12 months | Renal function stable,  Decreased proteinuria,  CRP gradually returns to normal. |
|  |  | 55/M | etanercept | TNFα biologics | Rectum | AS | 12 months | Clinical symptoms improved,  Proteinuria decreased,  Decrease in CRP. |
|  |  | 51/M | etanercept | TNFα biologics | Rectum | AS | NA | Renal function stable,  Decreased urine protein,  Inflammatory substances' level reduced. |
| 10 | Miyagawa, S., et al. (2020). | 57/F | etanercept | TNFα biologics | Heart, GI tract | RA | 60 months | Diastolic function improved at 4 years but failed at 5 years. |
| 11 | Serratrice, J., et al. (2003). | NA | etanercept | TNFα biologics | Kidney | Adult-onset Still's disease | 17 months | Proteinuria decreased significantly to 1.15 g/d,  Serum creatinine decreased to 1.76 mg/dL,  No side effects. |
| 12 | Alhalabi, M., et al. (2023). | 47/M | IFX，then UST | TNFα biologics，then IL12/23 biologics | Positive in kidney, negative in the colon and rectum | CD | 12 months | Clinical symptoms relieved,  Increased proteinuria,  SAA increased,  eGFR91.3mL/min/1.73 m2,  No side effects. |
| 13 | Fidalgo, C., et al. (2010). | 38/M | IFX，then ADA | TNFα biologics | Kidney | CD | 8 months | Clinical remission,  CRP decreased,  Proteinuria not improved, Renal function stable, Intestinal lesions not improve. |
| 14 | Eriksson, P., et al. (2021). | 60/M | IFX，then TCZ | TNFα biologics，then IL-6 biologics | Kidney | AS | NA | Renal function improved slightly,  SAA normal |
|  |  | 78/M | ADA，then TCZ | TNFα biologics，then IL-6 biologics | Kidney | AS | NA | Renal function improved slightly,  SAA normal |
| 15 | Vinicki, J. P., et al. (2013) | 48/F | ADA, then TCZ | TNFα biologics，then IL-6 biologics | Kidney | RA | 30 months | Clinical symptoms relieved,  Renal function recovery,  Proteinuria decreased,  CRP significantly decreased to normal level, No side effects. |
| 16 | Ravindran, J., et al. (2004). | 74/F | etanercept，then IFX | TNFα biologics | GI tract, kidney | RA | 24 months | Decreased proteinuria,  Renal function was stable,  Serum amyloid P decreased with radioiodine. |
| 17 | Chantarogh, S., et al. (2017) | 19/F | etanercept，then TCZ | TNFα biologics，then IL-6 biologics | Kidney | JIA | 12 months | Clinical symptoms relieved,  Renal function stable,  CRP significantly decreased to normal,  Slight decrease in IL-6. |
| 18 | Hattori, Y., et al. (2012) | 58/F | etanercept，then TCZ | TNFα biologics，then IL-6 biologics | Stomach and duodenum | RA | 50 months | Cardiothoracic ratio and left ventricular mass decreased significantly |
| 19 | Aït-Abdesselam, T., et al. (2010). | 26/F | Anakinra | IL-1 biologics | Kidney | CAPS | 60 months | Clinical symptoms relieved,  Proteinuria decreased significantly and maintained below 0.8g/d, CRP and SAA decreased to normal levels,  Serum creatinine stable at 0.62mg/dL. |
| 20 | Gentileschi, S., et al. (2017). | 59/F | Anakinra | IL-1 biologics | Kidney | TRAPS | 20 months | Clinical symptoms relieved,  SAA and CRP decreased,  Proteinuria decreased,  Primary diseases stable,  eGFR basically unchanged. |
| 21 | Kallianidis, A. F., et al. (2016). | 62/F | Anakinra | IL-1 biologics | Colon and kidney | HIDS | 9 months | No clinical response |
| 22 | Moser, C., et al. (2009). | 30/M | Anakinra | IL-1 biologics | Kidney, GI tract | FMF | NA | Clinical symptoms relieved. Decreased inflammatory markers. |
| 23 | Nalcacioglu, H., et al. (2018). | 16/F | Anakinra | IL-1 biologics | Retroperitoneal mass | FMF | 12 months | Clinical symptoms relieved, Decreased proteinuria, Normal Renal function, with creatinine 0.6 mg/dL |
| 24 | Stankovic Stojanovic, K., et al. (2012). | 55F | IFX，then Anakinra | TNFα biologics，then IL-1 biologics | Salivary glands, intestinal tract | FMF | 17 months | Clinical symptoms partially improved, Primary disease relieved, CRP decreased to normal, Renal function stable,  Proteinuria 520 mg/mmolC. |
|  |  | 27M | Anakinra | IL-1 biologics | Colon | FMF | 4 months | Clinical symptoms relieved, CRP and SAA levels returned to normal, Neutropenia and mild infection were observed. |
|  |  | 27F | Anakinra | IL-1 biologics | Heart | FMF | NA | Clinical symptoms relieved,  Primary diseases improved,  CRP decreased significantly,  Cardiac function recovered significantly. |
|  |  | 61F | Anakinra | IL-1 biologics | NA | FMF | 6 months | Clinical symptoms relieved,  CRP decreased significantly, No significant side effects. |
| 25 | Tastekin, F., et al. (2024). | 85/F | Anakinra | IL-1 biologics | Salivary glands, rectum | Jugular paraganglioma | 48 months | Clinical symptoms relieved,  CRP and SAA decreased,  Renal function and proteinuria were stable. |
| 26 | Roldan, R., et al. (2008) | 7/NA | Anakinra | IL-1 biologics | NA | FMF | 6 months | Clinical symptoms relieved and primary diseases controlled,  Inflammatory markers rapidly decreased to normal levels, No side effects. |
| 27 | Allinovi, M., et al.(2024) | 37/M | Anakinra | IL-1 biologics | Kidney and periumbilical fat | FMF | 27 months | Clinical symptoms relieved,  Proteinuria completely normalized (0.08 g/ day at last follow-up),  Renal function stable, Hypertension and neutropenia developed. |
| 28 | Bilginer, Y., et al. (2010) | 8/F | Anakinra | IL-1 biologics | Kidney | FMF and Bechet’s disease | 18 months | At 12 months: clinically symptomatic patients, inflammatory markers decreased to normal, proteinuria decreased. At 18 months: progressive increase in proteinuria (2.4 g/ day) and decrease in albumin level (26 g/L). |
| 29 | Goto, T., et al. (2023). | 57/M | canakinumab | IL-1β biologics | Kidney | FMF | 1 months | Clinical symptoms relieved,  Normal CRP,  Decreased SAA,  Decreased proteinuria. |
| 30 | Scarpioni, R., et al. (2015). | 52M | canakinumab | IL-1β biologics | Kidney, colon | Muckle-Wells syndrome | 18 months | Clinical symptoms relieved,  SAA and CRP decreased significantly,  Proteinuria significantly reduced, eGFR significantly improved, No side effects. |
|  |  | 26M | canakinumab | IL-1β biologics | Kidney | Muckle-Wells syndrome | 18 months | Clinical symptoms relieved, Proteinuria decreased significantly, eGFR significantly increased, CRP and SAA decreased significantly and maintained normal levels. |
| 31 | Gupta, A., et al. (2020) | 10/M | TCZ | IL 6 biologics | Kidney | JIA | 12 months | Systemic symptoms improved;  Proteinuria decreased significantly; Inflammatory marker ESR decreased significantly. |
| 32 | Sato, H., et al. (2009) | 53/F | TCZ | IL 6 biologics | Colon and stomach | RA | 4 months | Systemic symptoms improved;  CRP decreased to normal;  Re-biopsy of the GI tract showed improved SAA deposition. |
| 33 | Yamagata, A., et al. (2017) | 67/F | TCZ | IL 6 biologics | Kidney, colon, duodenum | RA | 9 months | Systemic symptoms improved;  Proteinuria decreased significantly;  SAA decreased to normal levels;  Repeated intestinal biopsies indicated a reduction in SAA. |
| 34 | Jung, J. Y., et al. (2021). | 66/F | TCZ | IL 6 biologics | Colon | RA | NA | Clinical symptoms relieved. |
| 35 | Rodrigues, F., et al. (2020). | 33/M | TCZ | IL 6 biologics | Kidneys, duodenum, and rectum | MKD | 12 months | Clinical symptoms relieved. |
| 36 | Seneschall, C., et al. (2024). | 79M | TCZ | IL 6 biologics | Kidney |  | 4 months | Clinical symptoms were relieved;  CRP and SAA returned to normal. |
| 37 | Nakamura, T., et al. (2014). | 70/F | Abatacept | Inhibitors of the CD80/ CD86-CD28 costimulatory pathway | NA | RA | NA | Clinical symptoms were relieved;  Re-biopsy of the GI tract showed regression of amyloid deposits. |
|  |  | 65/F | Abatacept | Inhibitors of the CD80/ CD86-CD29 costimulatory pathway | NA | RA | NA | Renal function remained stable;  Proteinuria did not improve; Re-biopsy of the GI tract was still positive. |
| 38 | Karadeniz, H., et al. (2020). | 24/M | canakinumab，then tocilizumab，then IFX，then tofacitinib | IL-1β biologics，then IL-6 biologics，then TNFα biologics，then JAK biologics | Kidney | FMF | 3 months | Proteinuria decreased;  Underlying disease remained stable;  No side effects. |

IFX: infliximab, ADA: adalimumab, UST: Ustekinumab, TCZ: tocilizumab, CD: Crohn's Disease, RA: rheumatoid arthritis, FMF: familial mediterranean fever, NA: not available, AS: Ankylosing Spondylitis, TRAPS: Tumor necrosis factor receptor-associated periodic syndrome, CAPS: cryopyrin-associated periodic syndrome, MKD: mevalonate kinase deficiency, HIDS: Hypergammaglobulinemia D and periodic fever syndrome, JIA: Juvenile idiopathic arthritis, SAA: Serum Amyloid A, CRP: C-reactive protein, F: female, M: male, GI: Gastrointestinal.

Table 2b Evidence of Therapeutic Efficacy of Biologics in AA Amyloidosis Treatment: Observational Studies and Systematic Review

| **#** | **Author (Year)** | **Biologics** | **Type of Biologics** | **Sample Size** | **Follow-up Duration** | **Etiology** | **Primary Outcomes** | **Literature Type** |
| --- | --- | --- | --- | --- | --- | --- | --- | --- |
| 1 | Esatoglu, S. N., et al. (2017). | IFX, ADA, ETN | TNFα biologics | 37 | 10 years (5.5-10.5) | Various (including AS, RA, FMF, CD, etc.) | Anti-TNF treatment can improve patient survival rates, especially when the baseline serum creatinine level is low or treatment is initiated early; Pay attention to serious adverse events such as infections. | Observational study |
| 2 | Keersmaekers, T., et al. (2009). | IFX | TNFα biologics | 4 | up to 8 years | Chronic inflammatory arthritis | The patients' clinical symptoms improved significantly; CRP and proteinuria decreased progressively with IFX treatment and remained at low levels in the long term; creatinine clearance rate did not change significantly. | Observational study |
| 3 | Nakamura, T., et al. (2010). | ETN | TNFα biologics | 14 | 90 weeks (79.75, 106) | RA | Proteinuria and serum albumin levels improved; SAA level decreased. | Observational study |
| 4 | Nakamura, T., et al. (2012). | ETN vs CYC | TNFα biologics | 62 + 24 (ETN) | 15 years | RA | ETN treatment was more effective than CYC treatment. | Comparative observational study |
| 5 | Pamuk Ö, N., et al. (2016). | NA | Primary TNFα biologics | 30 | 31.9 ± 34.3 months | AS and RA | It was effective in at least half of the patients; tuberculosis was the most important safety concern. | Observational study |
| 6 | Ortiz-Santamaria V., et al. (2003) | NA | TNFα biologics | 6 | 18 ± 9.16 months | NA | Inflammatory substances decreased significantly initially and then rebounded slightly, but did not reach the baseline level; serum creatinine increased slightly, but remained below 2 mg/dl; proteinuria did not increase; 3 patients discontinued treatment due to serious adverse reactions. | Observational study |
| 7 | Lane, T., et al. (2017). | Anakinra | IL-1 biologics | 11 | Median treatment follow-up of 1.8 (1-7.6) years | NA | SAA decreased significantly; AA amyloid deposits remained stable. | Observational study |
| 8 | Rodrigues, F., et al. (2022). | NA | IL-1 biologics | 86 | NA | CAPS | It was effective. | Observational study |
| 9 | İlgen, U., et al. (2023). | Anakinra, canakinumab | IL-1 biologics | 4 | Minimum 24 months, maximum 66 months | Unknown | All patients benefited, with decreased SAA and CRP and increased albumin levels. No adverse reactions were observed. | Observational study |
| 10 | Trabulus, S., et al. (2018). | Canakinumab | IL-1β biologics | 9 | NA | FMF | Clinical symptoms improved, but there were no statistically significant differences in laboratory tests. | Observational study |
| 11 | Kuroda, T., et al. (2016). | ETN, TCZ | TNFα biologics, IL-6 bilogics | 28 (10 received biologics) | 15.5 person-years in the biologic group; 34.3 person-years in the non-biologic group | RA | It improved the DAS28-CRP score and decreased SAA levels; there was no significant change in mortality; it increased the risk of infection. | Observational study |
| 12 | Lane, T., et al. (2015). | TCZ | IL-6 biologics | 20 | 23 months | Chronic inflammatory disorders | SAA decreased significantly; amyloid deposits partially regressed. | Observational study |
| 13 | van der Hilst, J., et al. (2016). | Anakinra, TCZ, rilonacept | IL-1 biologics | 104 | NA | FMF | IL-1 was safe and effective for FMF patients who were non-responsive or intolerant to colchicine and could reverse proteinuria. | Systematic review |
| 14 | Mertz, P., et al. (2025). | TCZ | IL-6 biologics | 126 | NA | FMF | TCZ was effective for FMF patients with secondary AA amyloidosis who were resistant to colchicine and IL-1 inhibitors; it had good safety. | Systematic review |

IFX: infliximab, ADA: adalimumab, TCZ: tocilizumab, CAPS: cryopyrin-associated periodic syndrome, FMF: familial mediterranean fever, AS: Ankylosing Spondylitis, RA: rheumatoid arthritis, CD: Crohn's Disease, NA: not available, ETN: Etanercept, CYC: Cyclophosphamide, SAA: Serum Amyloid A, CRP: C-reactive protein.
